# Beneficiary Experiences and Perceptions of Pradhan Mantri Jan Arogya Yojana-Social Endeavor for Health and Telemedicine (PMJAY-SEHAT) Services in Kashmir: A Facility-Based Cross-Sectional Study

**DOI:** 10.7759/cureus.111282

**Published:** 2026-06-22

**Authors:** Shahroz Nabi, Mehvish A Khan, Ishrat Rashid, S. Muhammad S Khan, Mohammad I Pandit, Inaam-Ul Haq

**Affiliations:** 1 Department of Community Medicine, Government Medical College, Srinagar, IND; 2 Department of General Medicine, Sher-i-Kashmir Institute of Medical Sciences, Srinagar, IND

**Keywords:** ayushman bharat, beneficiary perceptions, financial protection, healthcare access, jammu and kashmir

## Abstract

Background

Ayushman Bharat - Pradhan Mantri Jan Arogya Yojana (PMJAY) aims to improve access to secondary and tertiary healthcare while reducing out-of-pocket expenditure in India. In Jammu and Kashmir, the scheme is implemented as the Pradhan Mantri Jan Arogya Yojana - Social Endeavor for Health and Telemedicine (PMJAY-SEHAT) with universal population coverage, while evidence on beneficiary experiences in the region remains limited.

Objectives

The objective of this study was to assess beneficiary-reported experiences with PMJAY-SEHAT services in selected empanelled hospitals of Kashmir, focusing on scheme awareness, accessibility of Arogya Mitra helpdesk services, PMJAY card generation, hospital admission processes, out-of-pocket expenditure, transportation support, hospital service ratings, and overall perception toward the scheme.

Methods

A facility-based cross-sectional study was conducted in 2021 for a period of six months among 155 PMJAY-SEHAT beneficiaries and their attendants across selected public and private empanelled hospitals in Kashmir. Data were collected through face-to-face and telephonic interviews using a structured questionnaire. Descriptive statistics were used to summarise findings, and associations were assessed using the chi-squared test and Spearman's rank correlation.

Results

Among participants, n=113 (73%) were aware of PMJAY-SEHAT before hospital admission, and 86% reported easy access to the Arogya Mitra helpdesk. Most beneficiaries (n=124, 80%) did not incur out-of-pocket expenditure, while n=31 (20%) reported expenses primarily related to medicines. Transportation support was limited, with n=146 (94%) arranging transport independently. Admission processes were efficient, with n=96(80%) of patients admitted within 30 minutes. Overall satisfaction was high, with n=96 (62%) rating hospital services as excellent and 28% as good. A strong positive correlation was observed between hospital service ratings and overall perception of the scheme (ρ = 0.90, p < 0.05).

Conclusions

PMJAY-SEHAT has improved access to hospital-based care in Kashmir with high beneficiary satisfaction and limited financial burden. Addressing gaps in medicine availability and transportation support may further strengthen the scheme's effectiveness and financial protection.

## Introduction

Affordable and quality healthcare continues to be a major challenge in India, which is mostly due to high out-of-pocket spending (OOPE), which puts a huge burden on households in terms of financial strain [1]. It is estimated that almost 72% of OOPE is spent on primary healthcare services, which highlights structural discrepancies in public financing and service provision [2]. Global and national health policy discourse has long been identified with these issues. According to the Alma-Ata Declaration of 1978, primary healthcare was recognized as the cornerstone of the realization of Health for All, with equity, accessibility, and community-based care as principles of health systems empowerment [3]. The involvement of India in Universal Health Coverage (UHC) has also been reflected in some policy milestones. There was an early recognition that a unified and publicly funded health system was needed, as early as the Bhore Committee (1946) recommendations pointed to the vision of a single health system, followed by the National Health Policies of 1983 and 2002 [4]. The National Health Policy 2017 also expanded the UHC agenda by giving a priority to enhance access to quality healthcare services and insurance against disastrous spending on health [5]. Regardless of these policy initiatives, financial risk insurance is not sufficient to cover a large percentage of the population [6].

The data from the National Sample Survey of 2004-2014 demonstrate that the number of households with catastrophic healthcare spending has risen by almost 10 % [7]. The reason behind this trend can be partly explained by the strong role of the private sector that provides over three-quarters of outpatient care and almost two-thirds of inpatient services in India [8]. At the same time, India is experiencing an epidemiological and demographic shift with the growing burden of non-communicable diseases, which is only exerting more pressure on expensive secondary and tertiary care [9]. These same forces have underscored the weakness of current funding systems and strengthened the argument about the necessity of massive publicly funded health insurance plans. The Government of India, in turn, introduced the Ayushman Bharat initiative in 2018, with the key element of it being the Pradhan Mantri Jan Arogya Yojana (PMJAY) [10]. PMJAY is designed to cover secondary and tertiary hospitalization of around 50 crore beneficiaries, which is in line with the Sustainable Development Goals and inclusive health coverage [11,12]. The scheme aims to cut OOPE by providing cashless treatment in empanelled public and private hospitals, which enhances access to the much-needed healthcare services at the same time reducing the financial burden [13].

PMJAY in the Union Territory of Jammu and Kashmir has been adopted as PMJAY-Social Endeavor to Health and Telemedicine (SEHAT), and it is universal in that all residents are covered regardless of socioeconomic status [14]. This broadened area differentiates PMJAY-SEHAT as applied in other areas and is a powerful policy intervention to solve local inequities in health care access [15]. Considering the geographical issues, the mixed-public-private healthcare environment, and historical differences in service access in Kashmir, the scheme can be instrumental in enhancing healthcare use and financial coverage [16].

Although PMJAY-SEHAT is a large-scale and significant policy, there is a lack of empirical support about how it is implemented on the ground in Kashmir. The current literature in the rest of India has mainly concentrated on enrollment trends, usage rates, or aggregate financial performance, whereas minimal attention has been given to beneficiary perspectives. Regarding the case of Kashmir, a significant deficiency exists in systematic data on the perception of the beneficiaries towards the accessibility, quality, and responsiveness of provided services based on PMJAY-SEHAT and the degree to which the scheme achieves its desired aim of decreasing OOPE. There is a paucity of information regarding the experiences of beneficiaries regarding hospital processes, support programs like those provided by Arogya Mitra, and unmet perceived coverage.

It is necessary to understand how beneficiaries perceive things to determine the actual efficacy of health insurance programs funded by the state. The perceptions of service quality, accessibility, and financial protection have a direct impact on healthcare-seeking behavior, scheme use, and the belief people have in health systems. The lack of region-specific evidence in Kashmir is one of the key study gaps that hinder the capacity of policymakers and administrators to optimize the implementation plans and tackle the problems of context-specificity.

The study fills this gap by evaluating the perception of the beneficiaries towards PMJAY-SEHAT in the selected districts of Kashmir. The study offers empirical evidence on how the scheme performs at the facility level because of its emphasis on healthcare access outcomes, service experiences, and financial protection through the lens of beneficiaries. The results will be used to programmatically improve changes, enrich the scanty regional body of literature on PMJAY-SEHAT, and guide evidence-based decision-making to enhance the implementation of universal health coverage programs in such environments.

Objectives of the study

The objectives of this study were to assess beneficiary perceptions of PMJAY-SEHAT in Kashmir, with specific emphasis on healthcare access, service delivery, and financial protection. The study aimed to evaluate beneficiaries' experiences with hospital-based services under the scheme and to identify perceived gaps in implementation that may affect utilization and satisfaction.

## Materials and methods

Study design

The study was conducted as a facility-based descriptive cross-sectional study to evaluate beneficiary-reported experiences with PMJAY-SEHAT, including access to services, service provision, and financial protection in selected districts of Kashmir. This descriptive cross-sectional design was appropriate for assessing beneficiary-reported experiences and perceptions at a single point in time after receiving services under PMJAY-SEHAT; however, it did not allow in-depth qualitative exploration of beneficiary experiences. The facility-based design enabled direct assessment of hospital-level implementation processes, including enrollment support, admission procedures, and service use under the scheme. Beneficiary experiences and perceptions were assessed through structured questionnaire items, including Likert-scale responses, and were analyzed quantitatively using descriptive statistics rather than formal qualitative analysis. This design is commonly used in health services research to investigate patient-reported outcomes and implementation-related attributes of public health programs.

Study setting

This was conducted in the Kashmir region of the Union Territory of Jammu and Kashmir, which is administratively divided into ten districts. The process of data collection was done in Srinagar, Baramulla, and Ganderbal. A total of 10 hospitals were selected for this study, which included six government and four private hospitals. These areas were specifically chosen because of the relatively large number of PMJAY-SEHAT empanelled government tertiary care hospitals and private healthcare providers in these areas. Also, the number of beneficiaries utilizing PMJAY-SEHAT services in these districts is higher, which is why they are appropriate to evaluate the implementation of the scheme at various levels of care and ownership structure in the healthcare system of the region.

Study participants

The population that was used in the study included patients and, where necessary, attendants who had availed healthcare services at the empanelled hospitals under PMJAY-SEHAT within the study period. This was done by including the attendants when the patients could not respond because of illnesses or other limitations. Participants were identified through the institutional permissions and beneficiary lists of the participating hospitals to identify eligible individuals. One hundred and fifty-five respondents who fit the inclusion criteria were recruited. The interviews were only done after informed consent was obtained and the participants were assured that their responses would only be used in the study.

Sample size and sampling technique

The sample size was estimated using the OpenEpi online sample size calculator (Open-Source Epidemiologic Statistics for Public Health, Version 3.01) [17]. The following formula was used:

$n=Z^2 p(1-p) / d^2$

where n represents the required sample size, Z represents the standard normal deviate at a 95% confidence level, p represents the projected frequency, and d represents the absolute precision. Since no prior region-specific estimate was available for beneficiary awareness or service experience under PMJAY-SEHAT in Kashmir, a projected frequency of 50% was used because it provides the most conservative estimate and yields the maximum sample size when the expected prevalence is unknown. An absolute precision of 8% was selected, considering the exploratory, facility-based nature of the study and feasibility constraints during data collection. A design effect of one was applied because the study did not involve cluster sampling or multistage sampling; therefore, no adjustment for clustering was required. Based on these assumptions, the minimum required sample size was 150. After allowing for an approximate 3% non-response rate, the final sample size was increased to 155 respondents. Convenience sampling was used to select hospitals and participants because PMJAY-SEHAT was in the early phase of implementation, and eligible beneficiaries were mainly available in selected empanelled hospitals with higher patient flow. This sampling approach supported feasible data collection from eligible service users but was not intended to generate population-level estimates.

Study tool and questionnaire development

The structured semi-open questionnaire designed especially for this study was used as a data collection method. The development of the questionnaire was based on the review of the published literature on the publicly funded health insurance programs in India, PMJAY operation guidance, and a past study that evaluated the experience of beneficiaries [18]. The questionnaire had questions about the sociodemographic factors, knowledge of PMJAY-SEHAT, service accessibility, experience with Arogya Mitra helpdesks, out-of-pocket spending, and general satisfaction with healthcare services. Subject experts in community medicine were consulted to review the questionnaire and make it relevant and understandable. The questionnaire was translated into Urdu and Hindi to enhance participant understanding and to ensure that respondents could answer the questions in languages commonly understood in the study setting. Before the data collection, slight changes were introduced to the wording and order of the questions. The questionnaire was reviewed by subject experts and pilot-tested before data collection; however, formal psychometric validation was not performed. Beneficiary experience and perception items were captured through structured questions and Likert-scale response categories, not through open-ended qualitative interviews or focus group discussions.

Data collection

The study was carried out between April 2021 and September 2021. The data were collected by direct face-to-face interviews within the hospital territory and telephonic ones in case of absence of face-to-face communication. The interview was done by trained investigators according to a standardized protocol to provide consistency in the data collection. Before each interview, trained investigators explained the purpose of the study, the voluntary nature of participation, confidentiality of responses, and the participants' right to decline or withdraw without affecting their access to healthcare services. For face-to-face interviews, written informed consent was obtained using signed consent forms. For telephonic interviews, verbal informed consent was obtained before starting the interview and was audio-recorded with the participant's permission. Precautions were undertaken to ensure confidentiality and anonymity, and participants were assured that refusal to participate would not affect their access to healthcare services.

Study variables

The analysis focused on input and process indicators related to PMJAY-SEHAT implementation. Quantitative variables included out-of-pocket expenditure during hospitalization, time taken for consultation and admission, ease of locating the Arogya Mitra helpdesk, access to food and accommodation services, and ambulance or transport support. Beneficiary perceptions of hospital service quality, satisfaction with hospital services, perceived responsiveness of healthcare workers, and overall perception toward PMJAY-SEHAT were assessed using structured questionnaire items and Likert-scale response categories. These variables were treated as beneficiary-reported quantitative/descriptive variables rather than qualitative data requiring thematic analysis. These variables were selected to evaluate beneficiary-reported access, service experience, financial protection, and perceived implementation of PMJAY-SEHAT.

Statistical analysis

The data were entered into Microsoft Excel 2019 (Microsoft Corp., Redmond, WA, USA) and analyzed using SPSS Statistics version 22 (IBM Corp., Armonk, NY, USA). Frequencies and percentages were used to summarise sociodemographic characteristics and key study variables. Beneficiary experiences, hospital admission experience, service quality, and overall perception toward PMJAY-SEHAT were assessed using structured questionnaire items, including Likert-scale response categories. These variables were analyzed descriptively using frequencies and percentages rather than through formal qualitative analysis. The chi-squared test was used to assess associations between categorical variables, including beneficiary characteristics, awareness, and expenditure patterns. The relationship between hospital service ratings and overall perception toward PMJAY-SEHAT was evaluated using Spearman's rank correlation coefficient. A p-value of <0.05 was considered statistically significant.

Ethical considerations

Ethical approval for this study was obtained from the Institutional Review Board of Government Medical College, Srinagar, under approval number IRBGMC-SGR/SPM/806. Administrative permission was also granted by the Director of Health Services, Kashmir. All participants were informed about the purpose and procedures of the study, and written or verbal informed consent was obtained before data collection. Participant confidentiality was maintained throughout the study, and all data were anonymized before analysis to protect individual privacy.

## Results

Participant characteristics

The sample comprised beneficiaries of PMJAY-SEHAT of various sociodemographic profiles. A total of 155 participants were recruited for this study. There was a good balance of both male and female study participants, and the respondents had different degrees of educational attainment, from no formal education to postgraduate education. The demographic profile of Kashmir was represented in the religious composition to a large extent. Beneficiaries received care at various categories of healthcare facilities, with a larger percentage receiving care in government hospitals than in private institutions. A variety of clinical indicators, such as medical and surgical cases, pediatric services, oncology-related treatment, and other health requirements, were presented in terms of hospital admissions. Table 1 provides a summary of the sociodemographic and clinical characteristics of the participants in the study.

**Table 1:** Sociodemographic and clinical characteristics of study participants (N = 155) The table summarizes the sociodemographic and clinical characteristics of the study participants, including gender, education level, religion, place of admission, and reason for admission. Data are presented as frequency (n) and percentage (%).

Characteristic	Category	n (%)
Gender	Male	68 (44.0)
	Female	87 (56.0)
Education level	No formal schooling	34 (22.0)
	Middle school	24 (15.5)
	High school	44 (28.4)
	Graduate	35 (22.5)
	Postgraduate	18 (11.6)
Religion	Muslim	144 (93.0)
	Hindu	4 (2.5)
	Buddhist	3 (2.0)
	Sikh	4 (2.5)
Place of admission	Government tertiary care hospital	95 (61.3)
	Government secondary care hospital	20 (12.9)
	Private hospital	40 (25.8)
Reason for admission	Surgical	38 (25.0)
	Medical	40 (26.0)
	Pediatric	20 (13.0)
	Oncology	7 (5.0)
	Other	50 (31.0)

**Figure 1:**
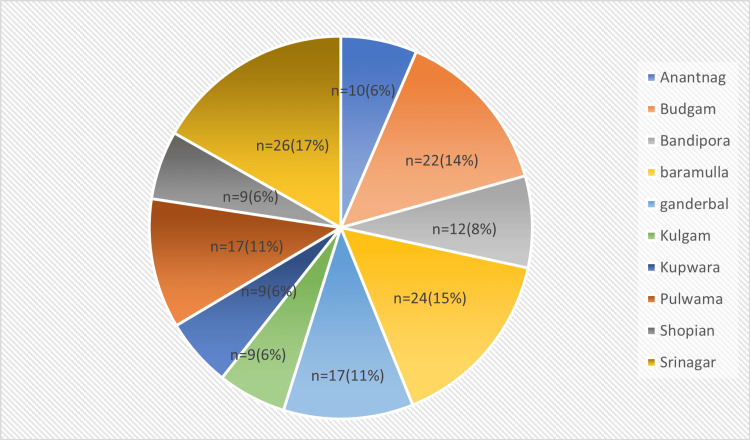
District-wise distribution of study participants The pie chart shows the distribution of study participants according to district of residence. Data are presented as frequency (n) and percentage (%).

The district-wise representation indicated that participants belonged to multiple districts of Kashmir, although the proportion of participants was higher from Srinagar, Baramulla, and Budgam. Although data collection was conducted in selected empanelled hospitals located in Srinagar, Baramulla, and Ganderbal, some beneficiaries sought treatment outside their home districts because empanelled services were concentrated in selected facilities. Figure 1 presents the district of residence of the beneficiaries rather than the district of the treating hospital. Since these data overlap with participant characteristics, the distribution of districts has been presented as a single figure with an explicit legend to prevent duplication.

Awareness of PMJAY-SEHAT and accessibility of services

Among the 155 beneficiaries included in the study, n=114 (73%) reported that they were aware of the PMJAY-SEHAT scheme before hospital admission, while the remaining participants became aware of the scheme during the process of seeking hospital care or card generation at the facility. Since all study participants were already beneficiaries who had accessed services under PMJAY-SEHAT, this finding should be interpreted as pre-admission awareness among scheme users rather than awareness in the general population. The support services related to the scheme were generally rated as easy to access, and n=133 (86%) of beneficiaries reported that the Arogya Mitra helpdesk was easy to locate at the hospital facility. There were different enrollment routes for PMJAY cards. Over 50% of respondents, n=87 (56%), acquired their cards at Khidmat Centers, while 12% acquired them at Common Service Centers. It is also worth noting that 18% of beneficiaries registered their PMJAY cards at the hospital upon admission, suggesting that facility-based enrollment systems played an important role in enabling timely access to services.

Association between gender and knowledge of PMJAY-SEHAT

Both the male and female participants were found to be aware of the PMJAY-SEHAT scheme, and no significant difference was observed in the knowledge level of these two groups. Statistical analysis revealed that there was no significant difference between gender and the knowledge about PMJAY-SEHAT (Chi-square test, p = 0.585). Table 2 displays the correlation between gender and scheme awareness.

**Table 2:** Association between gender and awareness of PMJAY-SEHAT Data are presented as frequency (n) and percentage (%). The association between gender and awareness of Pradhan Mantri Jan Arogya Yojana-Social Endeavor for Health and Telemedicine (PMJAY-SEHAT) was assessed using the chi-squared test. A p-value <0.05 was considered statistically significant, and a p-value <0.01 was considered highly significant.

Awareness of Pradhan Mantri Jan Arogya Yojana (PMJAY SEHAT)	Male (n = 68)	Female (n = 87)	Total (N = 155)	p-value
Aware	52	62	114	0.585
Not aware	16	25	41	

Out-of-pocket expenditure and service utilization

The majority of the beneficiaries stated that they have received care without spending out of pocket during their hospitalization, as part of PMJAY-SEHAT, whereas there have been some participants who had to spend on their medicines. Out-of-pocket spending was also found in various categories of health care institutions, such as government tertiary, government secondary, and private hospitals. The support for transportation was also minimal, where n=146 (94%) of the respondents organized transportation on their own, and those who offered transportation assistance were only n=9 (6%). Free food and accommodation were universally seen in the government hospitals, and accommodation support was intermittently seen in the private facilities. The statistical analysis has provided no significant correlation between out-of-pocket spending and service type used in medicine (p = 0.778). Besides, gender and PMJAY-SEHAT awareness did not show any statistically significant relationship (chi-squared test, p = 0.585). Table 3 provides the allocation of out-of-pocket expenditure on medical services according to categories.

**Table 3:** Association between out-of-pocket expenditure and type of medical service Data are presented as frequency (n) and percentage (%). The association between out-of-pocket expenditure (OOPE) and the type of medical service availed at empanelled hospitals was assessed using the chi-squared test. The chi-squared value was χ² = 1.094, with 3 degrees of freedom and a p-value of 0.778. A p-value <0.05 was considered statistically significant, and a p-value <0.01 was considered highly significant.

Type of service	OOPE present n (%)	No OOPE n (%)	Total
Surgical (n = 38)	30 (78.9)	8 (21.1)	38
Medical (n = 40)	35 (87.5)	5 (12.5)	40
Pediatric (n = 20)	17 (85.0)	3 (15.0)	20
Other (n = 57)	48 (84.2)	9 (15.8)	57
Total	130 (83.9)	25 (16.1)	155

Hospital admission experience and service quality

**Figure 2:**
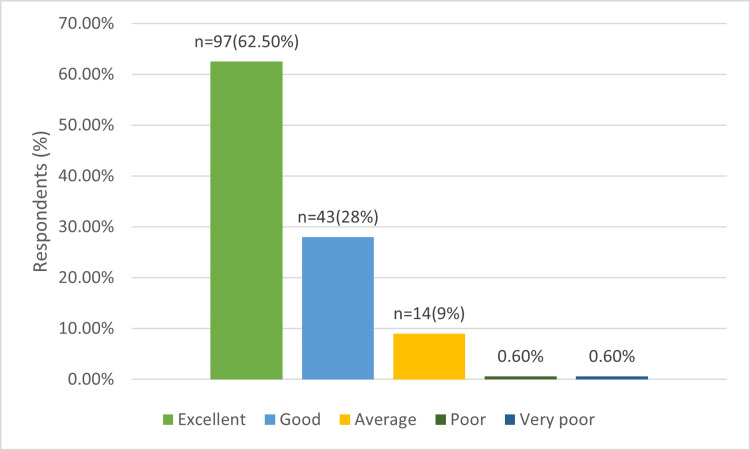
Beneficiary feedback on hospital services under AB-PMJAY The bar chart shows beneficiary feedback on hospital services under Ayushman Bharat Pradhan Mantri Jan Arogya Yojana (AB-PMJAY). Data are presented as frequency (n) and percentage (%).

Hospital admission experience and service quality were assessed using structured questionnaire items and Likert-scale response categories. The findings were summarised using frequencies and percentages. Most beneficiaries reported prompt admission after arrival, and only a small number reported concerns related to service delivery, staff conduct, or informal payment requests. Hospital service ratings were generally favorable, with most participants rating services as excellent or good. Figure 2 presents the distribution of beneficiary ratings of hospital services under PMJAY-SEHAT.

Perception of the PMJAY-SEHAT scheme and correlation analysis

**Figure 3:**
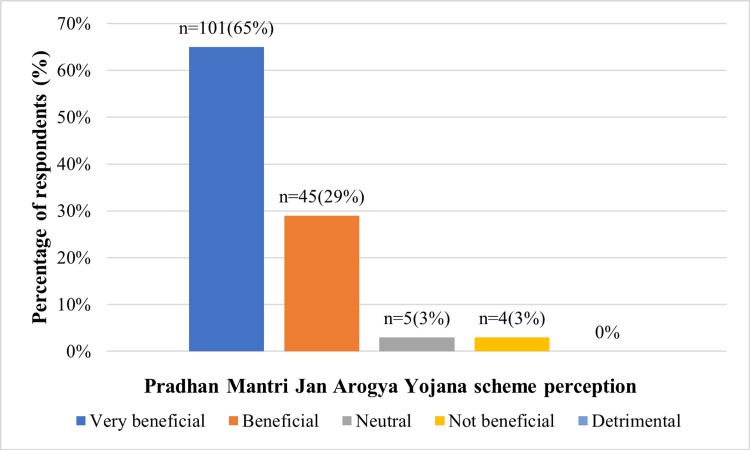
Beneficiary perception of the PMJAY-SEHAT program The bar chart shows beneficiaries' perceptions of the Pradhan Mantri Jan Arogya Yojana-Social Endeavor for Health and Telemedicine (PMJAY-SEHAT). Data are presented as frequency (n) and percentage (%).

The PMJAY-SEHAT scheme was viewed as favorable by the majority, with the majority of the respondents perceiving it as a positive platform, though a lesser proportion was neutral or unfavorable. Correlation analysis revealed that there was a strong positive correlation between perceptions of the quality of service provided by the hospital and perceptions of the PMJAY-SEHAT scheme, which means that the more positive the perceived service quality, the more positive the perceived PMJAY-SEHAT scheme. Figure 3 depicts the beneficiary perception towards the PMJAY-SEHAT scheme.

Table 4 shows a strong positive correlation between hospital service ratings and overall perception toward the scheme (Spearman's ρ = 0.90, p < 0.05).

**Table 4:** Correlation between hospital service ratings and overall perception toward the scheme

Variable	Spearman's correlation coefficient (ρ)	p-value	Interpretation
Hospital service ratings vs overall perception toward the scheme	0.90	<0.05	Strong positive correlation

A strong positive correlation was observed between hospital service ratings and overall perception toward the scheme (Spearman's ρ = 0.90, p < 0.05), indicating that participants who reported better hospital service experiences tended to have a more favorable perception of PMJAY-SEHAT.

## Discussion

This study reviewed the perceptions of beneficiaries of PMJAY-SEHAT and its role in enhancing healthcare access and financial security in Kashmir at the initial stages of implementation. The results provide valuable data on the functioning of a massive, publicly funded health insurance plan at the facility level in an area with complex geographic and administrative characteristics. By focusing on beneficiary-reported experiences in addition to utilization-related indicators, this study provides preliminary facility-level evidence on perceived service delivery and responsiveness under PMJAY-SEHAT; however, in-depth exploration of beneficiary experiences would require qualitative methods such as focus group discussions or interviews.

Among the most important findings of the study, a relatively high level of awareness of PMJAY-SEHAT among beneficiaries should be noted. This indicates that outreach and information dissemination systems have attained fair penetration, regardless of settings that are not easy to penetrate (poisonous terrain, inconsistent health literacy, and program implementation). The findings are relative to studies in other sections of India that have reported the level of awareness as being low, and therefore, the universal coverage model applied in Jammu and Kashmir could have promoted the level of visibility and recognition of the scheme [19]. Effective use of health insurance programs requires adequate awareness, as earlier assessments of publicly funded schemes indicated that the greatest barrier to access was a lack of knowledge about entitlements and procedures to access the programs [20].

The overall level of satisfaction amongst the beneficiaries of the hospital-based services of PMJAY-SEHAT was positive, as they reported positive opinions of the admission process, service delivery, and communication between them and medical workers [21]. The effective admission schedules and Arogya Mitra helpdesks seem to have played a large part in both access facilitation and administrative obstructions [22]. Another study on PMJAY in other states has shown similar results, with patient navigation proxying better with dedicated support staff in place, and patient waiting times decreasing [14]. The above observations support the need to reinforce the frontline implementation mechanisms to achieve the transformation of insurance coverage to meaningful access to services.

Although the general views are positive, the existence of out-of-pocket spending by a group of beneficiaries is an issue of concern [10]. Even though the majority of respondents indicated their hospitalization was paid in cash, some beneficiaries still paid money for medications, diagnostics, and non-medical expenses like transportation. This observation is in line with the past study on the health insurance schemes in India, which has indicated that the insurance scheme is not a sufficient solution to the financial burden. The existence of disparities in the supply of medicine, a shortage of diagnostic coverage, and indirect costs remain obstacles to achieving the goals of full financial insurance [23]. These problems cannot be resolved by insurance design alone, as hospital supply chains and ancillary services have to be improved.

The perceived quality of hospital service and the perception of the PMJAY-SEHAT scheme in general have a strong positive correlation, which indicates the key role of service delivery in building trust among the public’s perception of health programs [24]. The better the experiences in the hospital, the more inclined the beneficiaries were to make a positive assessment of the program, and it seems that the quality of care and patient-centered services are the key factors that define the acceptance of the program [13]. This connection supports the idea that any financial coverage should be supported by the quality assurance systems to maintain the long-term usage and trust in the publicly funded health systems [8].

Policy and programmatic-wise, the results indicate that PMJAY-SEHAT has achieved significant gains towards the enhancement of access to secondary services, as well as tertiary health care services in Kashmir [25]. Nevertheless, it should be refined to make it more effective through specific improvements. Increasing the supply of necessary drugs and tests in empanelled health institutions, enhancing transportation benefits to beneficiaries, and making reimbursements to medical institutions on time will assist in minimizing residual out-of-pocket spending and the avoidance of informal payments [21]. More equitable use of available resources can be done by further investing in educational efforts and streamlining admissions procedures, especially in those populations that have only limited previous access to formal healthcare systems.

In general, this study will offer region-specific data on PMJAY-SEHAT implementation and perceived effectiveness in Kashmir. It presents the results of success and ongoing challenges, pointing to the necessity of a combined approach to achieving the correspondence of insurance cover to the capabilities of the health system and the quality of care. The lessons learned during this study can be used in the future to amend the programs in Jammu and Kashmir and provide insights into such kinds of universal health coverage programs in other areas with similar health system limitations.

Limitations and future recommendations

There are several limitations of this study. The cross-sectional design captured beneficiary perceptions at a single point in time and did not allow assessment of changes over time or causal relationships. The use of convenience sampling may have introduced selection bias and systematic error, as beneficiaries who had successfully accessed PMJAY-SEHAT services at empanelled hospitals were more likely to be included. Therefore, the findings should be interpreted as facility-based beneficiary experiences rather than population-level estimates. Self-reported responses may also have been affected by recall bias and social desirability bias, particularly for out-of-pocket expenditure and satisfaction-related responses. The absence of a qualitative component, such as in-depth interviews or focus group discussions, limited the ability to explore beneficiary experiences and perceptions in greater depth. Future mixed-method studies are recommended to obtain broader and more detailed insights into beneficiary experiences after receiving services under PMJAY-SEHAT. The study also did not compare OOPE or service quality ratings between government and private hospitals, as the facility-based convenience sample was not designed or powered for hospital-ownership subgroup analysis. Future studies using stratified sampling by hospital ownership may provide more robust evidence on differences in financial protection and service quality across facility types. The study was conducted in selected districts of Kashmir, which may limit generalizability to other areas with different healthcare infrastructure and administrative contexts.

The PMJAY-SEHAT program in Kashmir requires targeted programmatic and system-level measures to address the remaining gaps identified in this study, particularly residual out-of-pocket expenditure, limited transport support, medicine availability, diagnostic access, grievance redressal, and consistency of service delivery across empanelled facilities. Availability of essential medicines and diagnostic facilities in empanelled hospitals should be improved to further reduce out-of-pocket expenditure and strengthen financial protection. Transport support should also be strengthened, especially for beneficiaries from remote areas, to reduce indirect costs related to accessing hospital-based care. Continued investment in hospital infrastructure, workforce capacity, and patient-centered services is needed to maintain service quality. Monitoring, accountability, and grievance-redressal mechanisms should be reinforced to support consistent implementation of the scheme. Future longitudinal and mixed-method studies are recommended to assess long-term outcomes and explore beneficiary experiences in greater depth. Future studies using stratified sampling across government and private facilities may also provide more robust comparative evidence on service quality and financial protection across hospital types.

## Conclusions

This study found that PMJAY-SEHAT beneficiaries in selected empanelled hospitals of Kashmir reported high awareness, favorable hospital service experiences, and generally positive perceptions toward the scheme. Most beneficiaries reported cashless care during hospitalization, suggesting financial protection under the scheme, although some continued to incur out-of-pocket expenditure, mainly for medicines and transportation. Hospital service ratings were positively correlated with overall perception toward PMJAY-SEHAT, indicating that beneficiary experience at the facility level may influence perceived scheme effectiveness. These findings suggest that PMJAY-SEHAT has contributed to beneficiary-perceived access and financial protection in the study setting, while residual expenditure and service-support gaps remain important areas for program improvement.
